# Trust your gut: the neuro-gastroenteric mechanics of prediction and expectation

**DOI:** 10.3389/fnhum.2026.1956182

**Published:** 2026-09-28

**Authors:** Alessia Caccamo

**Affiliations:** 1 Department of Mathematics and Statistics, University of Exeter, Exeter, United Kingdom; 2 Living Systems Institute, University of Exeter, Exeter, United Kingdom

**Keywords:** gut microbiome, gut-brain axis, hippocampus, nutritional psychiatry, predictive coding, prior expectations

## Abstract

Living organisms constantly make probabilistic predictions about what they expect to see in their external environment, and actively solicit information from the environment to inform their predictions. This process is foundational for many frameworks including Active Inference and Predictive Processing. It relies on organisms having prior expectations, entailed by an internal generative model of the environment. Posterior expectations are updated based on the selected data, driven by prediction error minimization. However, failures to contextualize the precision or confidence placed in predictions, can give rise to perceptual abnormalities that are at the basis of various psychiatric conditions. Different factors can influence this process. Notably, disruptions in the gut microbiome have been implicated in some psychotic disorders. Gut microbial dysregulation has also been associated with alterations in neurotransmitter synthesis, metabolism, and turnover, which may affect processes involved in the encoding of precision of prediction errors. On the basis of this gut-brain connection, this Hypothesis and Theory article hypothesizes that the gut microbiome plays a role as a modulator of predictive processing, influencing how predictions are formed and expectations updated. Specifically, this article explores the importance of “mental model" embodiment, and considers the role of hippocampal activity in predictive coding and its modulation by the gut microbiome. Finally, it offers a novel perspective on the use of cortico-hippocampal-gastroenteric frameworks for inferring personalized dietary interventions in nutritional psychiatry.

## 1 Introduction

Predictive processing is a theory of brain function, where perception is cast as a process of generating predictions about sensory inputs and updating those predictions in response to prediction errors. Predictive coding provides a neural implementation of this process, and explains how neural systems may support perception and inference, through hierarchical prediction and prediction-error signaling. These ideas can be traced to the 19th century, with roots in philosophical theories of perception and the idea of ‘unconscious' inference (Helmholtz, 1866). In 1999, predictive coding entered the field of cognitive neuroscience through the seminal work of Rao and Ballard (1999). It was subsequently extended by Professor Karl Friston to account for the free energy principle and empirical Bayes (Friston, 2003; Friston et al., 2006), where the free energy principle provides a general formal framework for understanding inference and action. Predictive coding has since become an influential framework for explaining psychiatric conditions (Friston, 2022), offering mechanistic explanations for pathological belief updating.

Computational implementations of predictive coding provide powerful tools for investigating (patho)physiological mechanisms and for identifying computational biomarkers of clinical relevance. For instance, different psychiatric conditions, including schizophrenia, depression, anxiety, and attention deficit hyperactivity disorder (ADHD), involve aberrant prediction error signaling or precision-weighting, and exhibit different “attractor" dynamics (Friston and Kiebel, 2009) related to predictive coding. These may lead to the formation of false beliefs (psychosis), maintenance of rigid negative beliefs (depression), stability of maladaptive predictions (anxiety) (Shaw et al., 2026), or increased reliance on sensory inputs (ADHD; Gonzalez-Gadea et al., 2015).

While these computational advances have contributed to elucidating pathological mechanisms, psychiatric disorders present other types of clinical and societal challenges. For instance, persistent “treatment gaps” have been reported internationally, varying substantially by disorder, healthcare setting, and country (Kohn et al., 2004; Roberts et al., 2022; Barbui et al., 2025). In England, these are recognized at the policy level, where key priorities include reducing inequalities, improving timely access to support, increasing funding for services, and increasing the availability of trained mental health professionals (Department of Health and Social Care, 2026a). Within this broader agenda, disorders such as ADHD have received increased attention. However, similar challenges affect, more broadly, all neuropsychiatric conditions, including schizophrenia (Cooper et al., 2026).

Advances in gut–brain neuroscience and brain–body research may offer a valuable framework for identifying novel and accessible treatment approaches to address this gap. The gut microbiome, and its links to neurological functions, have emerged as key areas of biomedical research and have gained popularity across gastroenterology and neuroscience (Valdes et al., 2018; Asnicar et al., 2026; Jacka, 2017; Cryan et al., 2019). As the gut is a modifiable determinant of brain health, dietary interventions are promising strategies for the management of neuropsychiatric conditions (Jacka et al., 2017; Fackelmann et al., 2025). Dietary interventions are specifically attractive due to cost-effectiveness, non-invasiveness, and may, in certain contexts, present lower implementation barriers than pharmacological interventions, particularly with respect to regulatory requirements and the risk of drug-specific adverse effects. This would be contingent on the nature of the intervention and its intended use. For instance, antipsychotic medications, can cause cardiovascular complications, movement disorders, and metabolic disturbances.

Dietary interventions may offer a complementary strategy to mitigating adverse effects by reducing reliance on high-dose pharmacological treatment. They may also be translated into clinical practice more quickly compared to novel pharmacotherapies, which typically span decades from discovery to approval. Moreover, for some microbiome-based interventions there may be alternative regulatory pathways that facilitate clinical implementation without requiring a microbiome-based medicinal product (Department of Health and Social Care, 2026b). Ultimately, novel strategies that target the microbiome through theory-driven pathways could complement existing therapeutic strategies, while also representing a potentially modifiable factor in the risk of developing psychiatric conditions.

Therefore, this Hypothesis and Theory article provides a mechanistic understanding of the relationship between brain function and gut health, proposing that cortical predictive processing is dependent on the modulation of hippocampal function by the gastrointestinal tract, with effects on the precision-weighting of prediction errors. While this article focuses specifically on psychotic manifestations in schizophrenia, the proposed hypothesis is also translatable to other neuropsychiatric conditions. Previous work has incorporated gut microbial dysbiosis into predictive and interoceptive models of affective symptoms (e.g., Büttiker et al., 2021). Moreover, gut microbiota–hippocampus interactions and microbiota-targeted interventions have been proposed as potential avenues for understanding and treating cognitive impairments in emerging psychosis (Iseli et al., 2023). A substantial body of work has linked the gut microbiome to brain function and psychiatric conditions (e.g., Berding et al., 2021; Zheng et al., 2019; Naidoo, 2020), as well as hippocampal function to microbial activities (e.g., Sarubbo et al., 2022; Liu et al., 2023; Alavian and Safaeian, 2025) and predictive processes (Barron et al., 2020). Building on these established concepts from predictive coding and microbiome–brain research, the present work provides a unified and operational framework in which gut microbiota influences hierarchical predictive coding, including the contribution of hippocampal mechanisms to the propagation of prediction errors across cortical hierarchies, in both health and disease. Specifically, it highlights a role for the hippocampus in the retrieval of memory representations that provide experience-dependent priors, and in the precision-weighting of prediction errors that drive learning and the updating of prior expectations.

The article provides a theoretical background to predictive coding and the gut-brain axis and introduces this unified framework that couples brain function—specifically, cortical hierarchies and hippocampal contributions to the encoding of priors and prediction error weighting—to bodily processes, such as microbial signaling in the gut. Finally, it discusses future prospects stemming from this framework. For instance, guiding computationally informed dietary interventions or nutraceutical approaches—the use of specific food-derived bioactive compounds or products—used in adjunction to pharmacotherapy, for the management of psychiatric conditions and for supporting broader aspects of brain health.

## 2 Generative models of the brain

The “Bayesian brain” hypothesis (Helmholtz, 1866; Friston and Price, 2001; Doya et al., 2007) draws upon a long history of considering the brain as a predictive “machine.” According to this hypothesis, the brain generates predictions and makes inferences on the external environment by combining prior expectations (the “mental model”) with sensory data.

Active Inference and Predictive Coding principles arose from this perspective and operate formally according to Bayesian mechanics. Active Inference is the framework that characterizes Bayes optimality in living systems (Parr et al., 2022), where Bayes optimality refers to inferring states of the world based on sensory observations—and selecting those observations that enable an inference to the best explanation. In living organisms, this can be understood in terms of minimizing surprise or, equivalently, maximizing model evidence (a.k.a., marginal likelihood), with variational free energy providing a tractable upper bound on surprise (while its negative provides a lower bound on the log model evidence). This leads to a view of active engagement with the world as a process of self-evidencing (Hohwy, 2025), understood as the minimization of surprise and uncertainty, with (precision-weighted) prediction errors providing the basis for updating predictions about sensory inputs. The brain handles prediction errors either by changing its internal beliefs—i.e., updating prior expectations to posterior expectations—to match the sensory data, or by actively creating the sensorium. This means fulfilling predictions, and sampling the environment to self-evidence, i.e., to make sensory input conform to prior expectations. In summary, predictive coding is the mechanism for perceptual inference that enables belief updating, while active inference incorporates action into the inferential process, enabling organisms to realize predictions based on their updated beliefs.

From a mathematics perspective, prior expectations are part of a generative model; namely, a probabilistic mapping between hidden causes and observed consequences (the sampled data). The generative model is defined as the joint probability of the likelihood of observing the data given the causes, and prior beliefs about the causes. As surprise cannot generally be computed directly, under specific assumptions it can be approximated using variational free energy, which provides an upper bound on the negative log probability of observing the data under the generative model (the marginal probability). Organisms can pursue surprise minimization through variational free energy minimization, and a good generative model is one that renders the sensory exchange with the world as likely as possible. Please note that a generative model represents the internal model of an organism, encoding its beliefs about what could generate the sensory data, whereas the generative process involves the physical dynamics of the organism, its body, and its environment, that generate those data. This theoretical formulation provides a means for understanding information processing in the brain, and it has since been applied to the study of physiological and pathophysiological brain function, through the use of modeling approaches such as Dynamic Causal Modeling (DCM) (Friston et al., 2003). These applications include the study of sensory processing, learning, and attention, as well as neuropsychiatric conditions such as schizophrenia, or substance-induced psychosis. Interestingly, psychosis is thought to emerge from disruptions in prediction error minimization, including abnormalities in the precision weighting of sensory evidence and prior beliefs.

Technically, precision reflects the confidence or credence afforded to prediction errors. In hierarchical generative models, the relative precision of prediction errors at lower (e.g., sensory) and higher (e.g., prior) levels can have a profound effect on belief updating and subsequent active inference. In brief, if sensory precision is too high, prior beliefs fail to contextualize sensory data as if the brain is affording too much attention to sensory information, relative to its experience-dependent prior beliefs. Conversely, if sensory precision is too low, prior beliefs predominate and are no longer constrained by sensory evidence. This can lead to failures of inference, such as inferring something is there when it is not (i.e., Type I statistical errors that may provide a good explanation for hallucinations and delusions in schizophrenia). Conversely, the brain may infer something is not there when it is (i.e., Type II statistical errors that may provide a collection for neglect, agnosia, and dissociative syndromes).

Please see Friston (2022) for a review of the underlying pathophysiology that turns upon the encoding of precision in predictive coding schemes. This is usually associated with the postsynaptic gain (c.f., cortical gain control or excitation-inhibition balance) of neuronal populations encoding predictions and prediction errors. A key determinant of the implicit encoding of certainty or precision is the balance of excitatory and inhibitory mechanisms in cortical microcircuits that turn on GABAergic function; e.g., the modulation of fast-spiking inhibitory interneurons and their interactions with excitatory pyramidal cells which broadcast predictions and prediction errors. In what follows, we look at a particular (extra-neuronal) source of this modulation.

## 3 The gut-brain axis

The gastrointestinal (GI) tract has long been regarded as central to human health. Its importance was first acknowledged in a statement often attributed to Hippocrates suggesting that “all disease begins in the gut” (Lyon, 2018), and subsequently reintroduced in the longstanding traditions of Ayurvedic and Chinese medicine. However, it has only recently gained prominence in modern healthcare, driven by growing evidence establishing the role of the gut microbiome in human physiology and its potential as a therapeutic target. Indeed, the GI tract is populated by a large ecosystem of microorganisms, such as bacteria, archaea, protozoa, and fungi—forming the microbiota—that are responsible for nutrient metabolism, fermentation of non-digestible dietary fibers, and immune responses. The microbiome represents the collective genomes of these microbes (Valdes et al., 2018). It is 100 to 150 times larger than the human genome and significantly affects the health, fitness and phenotype of the human host. For instance, gut microbes can synthesize psychoactive metabolites from nutrient precursors, affecting cell signaling in a way that is comparable to hormones (Ezra-Nevo et al., 2020).

While heritable factors influence the microbiome, recent evidence suggests that it is predominantly shaped by diet (Asnicar et al., 2026). Diet can influence microbiota diversity and is associated with distinct health markers. Microbiota diversity contributes to a more resilient ecosystem, where functionally-related species compensate for one another, maintaining metabolic stability. This diversity is influenced by dietary habits, including the intake of protein, fats, carbohydrates, and fiber, as well as by the range of plant food consumption (Fackelmann et al., 2025). Conversely, food additives and artificial sweeteners, commonly found in highly processed foods, negatively affect the microbiome and promote inflammatory responses that deplete the microbiome (Valdes et al., 2018). A lower number of microbial species and dysbiosis—that is defined as an imbalance in microbial community diversity, microbiota composition (number and type), or microbiome function (metabolic and biological activities and interactions)—are typically associated with the emergence of diabetes, obesity and several other conditions (Valdes et al., 2018; Bull and Plummer, 2014).

Notably, the gut and the central nervous system communicate through complex multidirectional interactions, referred to as the gut-brain axis (GBA) (Berding et al., 2021; Gwak and Chang, 2021; Loh et al., 2024). Gut-brain communication is facilitated by physical pathways, such as the vagus nerve, or chemical pathways involving neurotransmitters. This GBA communication was further extended into a microbiome-GBA framework, where gut-brain communication is regulated by microbial species. This can involve intestinal permeability and the activity of the enteric nervous system, that is the network of neurons embedded in the walls of the GI tract. Microbiome-GBA communication can also involve immune responses, and the hypothalamic–pituitary–adrenal (HPA) axis—a neuroendocrine stress system that is functionally connected to the limbic system and that contributes to the regulation of stress responses, emotional processing, and memory function. Finally, the microbiome is also a critical regulator of hippocampal neurogenesis (Guzzetta et al., 2022; Sarubbo et al., 2022), considering that the hippocampus retains the capacity for the production of new neurons throughout life.

Many effects of gut microbiota on brain function are strain specific (Carabotti et al., 2015), such that diet-induced microbial depletion can cause a range of neurological and psychiatric conditions, including anxiety (Naidoo, 2020), depression, and schizophrenia (Clapp et al., 2017). Specific microbial strains have been implicated in the onset, progression, and pathophysiology of schizophrenia (Munawar et al., 2021; Tsamakis et al., 2022), through mechanisms, including the modulation of pro-inflammatory cytokine production, fatty acid and vitamin B6 metabolism, and microglial activation (Tsamakis et al., 2022). Supporting this association, transplantation of fecal microbiota from patients with schizophrenia into mice was shown to induce schizophrenia-like phenotypes accompanied by altered hippocampal levels of glutamate, glutamine, and gamma-aminobutyric acid (GABA) (Zheng et al., 2019).

These findings provide some insight into the neurochemical mechanisms linking gut microbiota and brain dysfunction, specifically psychosis. These mechanisms are pertinent to the development of theoretical models that can identify novel causal pathways underlying pathophysiology, and thus targets where therapeutic interventions may be effective.

## 4 Regulatory effects of the gut microbiome on model predictions

Thus far, this paper has established the conceptual foundations of predictive processing and predictive coding and introduced the influence of the gut microbiome on brain health and function. Notably, dysregulation of both systems has been implicated in psychiatric conditions, suggesting a putative functional interaction.

Specifically, the hippocampus is recognized to serve a key role in nutritional psychiatry and to play a role in the processing of prior expectations and prediction errors, as explained below. The remainder of this article will thus triangulate hippocampal function, predictive coding and gut-brain interactions. In so doing, it will propose a novel hierarchical predictive coding architecture that sees the gut microbiome as a key regulator of predictive processing.

### 4.1 The hippocampus in hierarchical predictive coding circuits

The canonical microcircuit model of predictive coding proposes that a cortical column contains the fundamental circuitry required to implement predictive computations. It comprises superficial (layers 2/3) and deep (layers 5/6) pyramidal neurons, alongside a pool of inhibitory interneurons responsible for maintaining excitation–inhibition (E/I) balance within the circuit (Douglas and Martin, 1991; Bastos et al., 2012). At a broader systems level, predictive coding can be conceptualized as a hierarchical framework, that is implemented through cortical circuits with functionally distinct populations that mediate top-down predictions and bottom-up prediction error signals (Kanai et al., 2015).

Within the cortical hierarchy, higher-order cortical levels generate top-down predictions of the representations encoded at lower levels of the hierarchy (Figure 1). These predictions are conveyed by deep-layer pyramidal neurons to adjacent lower cortical areas. In lower cortical levels, predictions are compared to the current representation generated from sensory inputs. Superficial pyramidal neurons encode the mismatch between predicted and observed representations, generating bottom-up prediction-error signals that are propagated to higher cortical levels to update internal representations. The reciprocal passing of top-down predictions and bottom-up prediction errors occurs recursively between adjacent levels of the cortical hierarchy, enabling minimization of prediction errors to construct an explanation of sensory inputs.

**Figure 1:**
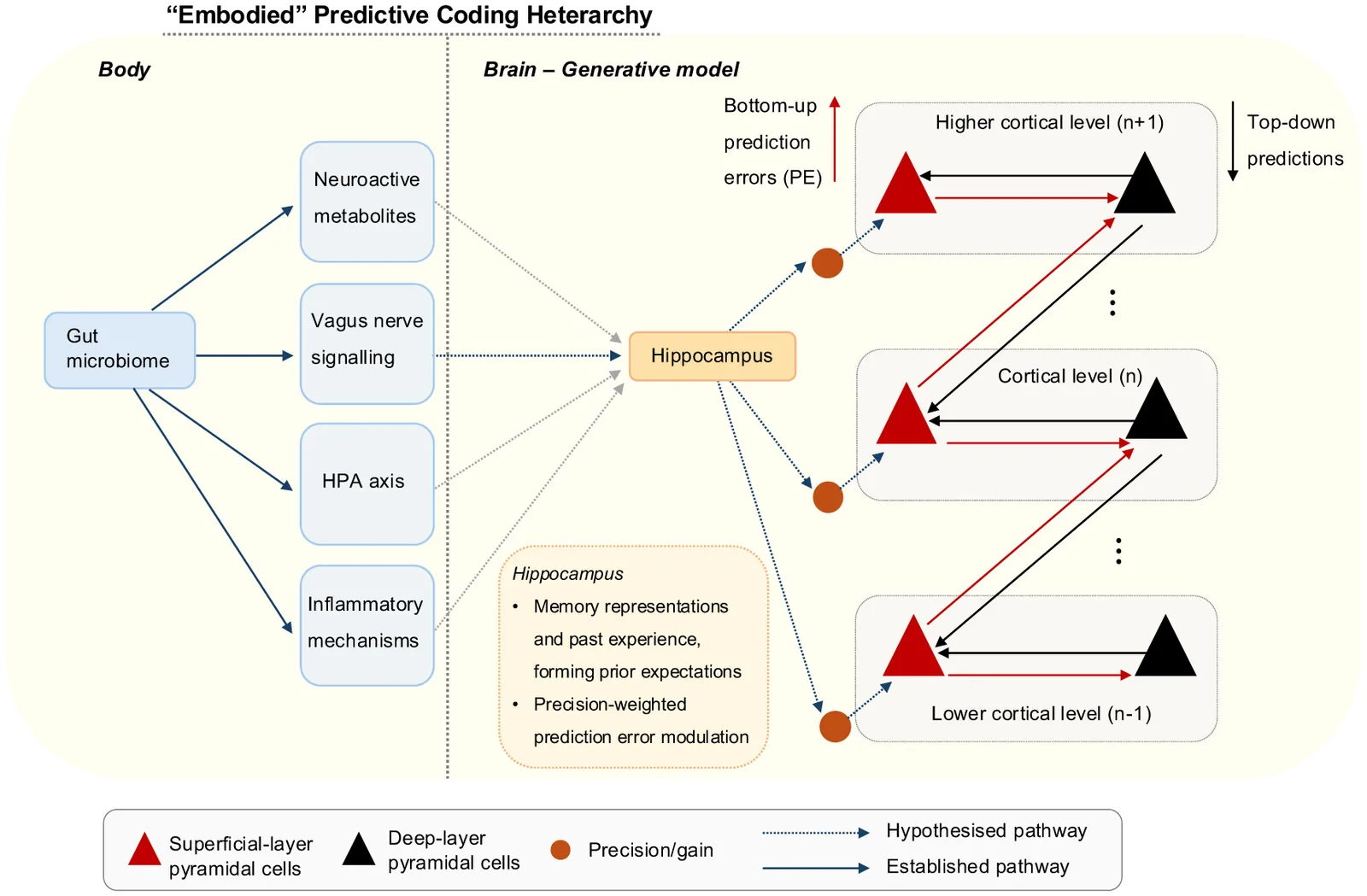
Hippocampus-mediated effects of the gut microbiome on predictive coding. The gut microbiome can communicate with the hippocampus through four main pathways. In this work, greater emphasis is placed on gut–vagal signaling. By shaping memory-based priors and precision weighting, the hippocampus modulates excitatory–inhibitory (E/I) balance and the propagation of prediction errors across the cortical hierarchy (see Kanai et al., 2015 for information on the cortical architecture). The model is conceptualized as a heterarchy (rather than a strictly hierarchical system), between gut-derived signals, hippocampal processes, and cortical predictive dynamics. Hypothesized pathways are inidcated as dashed lines, known pathways as solid lines. Precision/gain, refers to the precision weighting of prediction errors, thought to be encoded by the synaptic gain. PE, prediction error; HPA, Hypothalamus-pituitary-adrenal (axis).

Prediction errors are weighted by their expected precision (or confidence) (Kanai et al., 2015). This determines the extent to which prediction-error signals influence higher cortical representations and regulates the relative weighting of sensory evidence and prior expectations. Precision weighting is thought to be implemented through neuromodulatory and local excitatory and inhibitory synaptic mechanisms that regulate the gain of superficial pyramidal neurons encoding prediction errors. Moreover, while canonical predictive coding frameworks have predominantly considered the architecture of cortical circuits, with several extensions accounting for subcortical structures (e.g., thalamus, cerebellum, and basal ganglia) (Friston and Herreros, 2016; Friston et al., 2017; Kanai et al., 2015; Parr et al., 2021), this article considers a cortico-hippocampal perspective. Indeed, it is plausible that the hippocampus contributes to prediction error weighting, by targeting cortical circuits to modulate GABAergic transmission. In turn, this would weight prediction errors according to the relative precision of prior expectations and sensory evidence, and contribute to the retrieval of memory representations that provide prior expectations.

The hippocampus is a brain structure nested within the medial temporal lobe of each cerebral hemisphere (Anand and Dhikav, 2012). It is part of the limbic system, playing a role in emotion processing, through interactions with amygdala, thalamus, hypothalamus and cingulate gyrus. It plays a central role in the organization, consolidation, storage, and retrieval of memories. The hippocampus is also one of the few brain regions exhibiting adult neurogenesis, which is important for the maintenance of memory functions (Kempermann et al., 2015). It also receives highly integrated information from widespread cortical systems, that contribute to supporting complex representations of experiences and contextual information. Because of its functional connection to cortical networks, it is plausible that the hippocampus contributes to perceptual inference, providing memory-based predictions and contextual priors to cortical networks, in line with existing hypotheses, and as explained below

Empirical studies support the idea that the hippocampus contributes to perceptual inference by providing experience-dependent (i.e., prior) predictions encoded as memories and by modulating the precision weighting of prior expectations relative to observed sensory data. In other words, it modulates the relative influence of prior expectations and novel sensory information signaled by prediction errors, thereby selectively driving learning and the requisite updating of internal models. Consistent with this view, hippocampal activity has been shown to affect the precision-weighted prediction error represented in the superior parietal cortex, suggesting that the hippocampus can signal prediction errors, and regulate the response of cortical circuits that process prediction errors (Rigoli et al., 2019) This proposed role of the hippocampus in predictive processing can be related to its established role in memory and learning (Kumaran and Maguire, 2006; Bein et al., 2020; Aitken and Kok, 2022). The hippocampus can support the retrieval and reinstatement of memory representations of prior experience. Therefore, from a predictive coding perspective, such representations can function as prior expectations, or predictive representations of the environment (Liu et al., 2023).

Therefore, memories could provide experience-dependent and context-dependent representations that constrain the processing of incoming sensory information. In addition to providing priors for predictive processing, the hippocampus could also inhibit cortical representations of prediction errors, or facilitate the reinstatement of cortical representations of prior experience, during memory recall, through E/I balance regulation, as proposed by Barron et al. (2020). The signaling of prediction errors to update prior representations can also be understood as a form of hippocampal-dependent learning. From this perspective, prediction errors may signal that experience-dependent memories (priors) need to be updated to improve future predictions. In other words, prediction errors interfere with stable memory representations and prompt their updating, through a process mediated by the hippocampus (Sinclair et al., 2021). Taken together, these considerations indicate that the hippocampus may contribute to perceptual inference and predictive processing, first, by retrieving memory representations that provide experience-dependent priors, and second, by regulating the relative influence of these priors against incoming sensory information. The latter would therefore relate to the precision-weighting of prediction errors, that may influence the extent to which they drive learning through the subsequent updating of internal models.

Ultimately, sensory information is filtered and weighted based on predictions generated from prior representations that determine the relevance of the received sensory data, and based on the expected precision. This process may be driven by the modulation of GABAergic transmission in cortical networks among inhibitory neurons lower in the hierarchy and inhibitory mechanisms that regulate the gain and precision of neural representations. As memories are known to be instantiated as balanced excitatory–inhibitory ensembles, stored representations are likely to function as prior expectations that constrain cortical predictive coding via the modulation of inhibitory interneurons at lower levels of the cortical hierarchy (Barron et al., 2020). Therefore, because of its role in constructing abstract and context-dependent memory representations, the hippocampus is conceptualized as occupying a relatively high level within the predictive coding hierarchy, although its exact computational role and hierarchical position remains to be elucidated.

### 4.2 Gastroenteric modulation of hippocampal function

While the gut microbiota affects brain function broadly, the hippocampus is particularly susceptible to its effects (Jacka et al., 2015; Sarubbo et al., 2022; Mayer et al., 2015; ELeung and Thuret, 2015; Coccurello, 2025; Tang et al., 2021; Kuijer and Steenbergen, 2023; Noble et al., 2017). A growing body of evidence indicates that the gut microbiota plays an important role in regulating hippocampal learning and memory, by influencing adult neurogenesis, BDNF expression and long-term potentiation (LTP), but also by affecting GABA expression (in the hippocampus, but also across the brain) and by inducing structural changes and effects on synaptic plasticity (Tang et al., 2021; Kuijer and Steenbergen, 2023; Alavian and Safaeian, 2025). Dysbiosis can impair hippocampal function (Kuijer and Steenbergen, 2023) via neuroinflammatory pathways and microglial activation, as well as by modulating synaptic transmission and hippocampal oscillatory activities (Calıskan et al., 2022). In both animal models and humans, dietary interventions known to alter the gut microbiota have been associated with changes in hippocampal volume, which may be related, in part, to neurogenesis (Kuijer and Steenbergen, 2023).

The effects of the gut microbiota on hippocampal learning and memory can be ascribed to four main pathways: (1) vagal signaling, whereby microbial signals are transmitted to the brainstem through the vagus nerve, before being relayed to the hippocampus to modulate E/I balance; (2) the HPA axis (endocrine route); (3) modulation of inflammatory responses; (4) production of neuroactive metabolites (metabolic route). These pathways are not mutually exclusive, rather, they are closely integrated, collectively contributing to microbiota-modulated hippocampal function (Kuijer and Steenbergen, 2023). Because hippocampal memory representations are also recruited to generate predictions about future events (Liu et al., 2023), it can be argued that microbiome-mediated alterations of hippocampal function may affect predictive processing via the four main pathways outlined above (Suarez et al., 2018; Bertollo et al., 2025; Crain et al., 2025; Guzzetta et al., 2022).

Moreover, in the previous section we have seen that the hippocampus affects the precision weighting of prediction errors (Barron et al., 2020), and that the encoding of precision is closely related to synaptic gain and E/I dynamics A plausible pathway may start with the synthesis of GABA by gut bacteria from dietary substrates, that is sensed through GABAergic receptors in the ENS (Belelli et al., 2025). Such sensing may influence vagal afferent activity through an anatomical pathway in which visceral signals are relayed through the brainstem and transmitted via an ascending multisynaptic pathways to higher-order neural networks in the central nervous system. GI vagal afferents terminate in the nucleus of the solitary tract in the caudal brainstem. From there, a multisynaptic pathway that travels through the medial septum to the hippocampus is physiologically implicated in hippocampal function (Suarez et al., 2018).

This article proposes that GABAergic signaling may modulate vagal afferent activity and—through potential downstream effects on hippocampal networks (Barron et al., 2020)—influence E/I dynamics in the predictive coding hierarchy (Figure 1). Such modulation could affect synaptic gain and the precision-weighting of prediction-error signals, providing a potential, but not exclusive, mechanism through which the gut microbiome influences predictive coding. Effect direction and magnitude may vary between individuals, as a function of differences in gut microbial composition, brain anatomy, neural dynamics and function. Moreover, future work should investigate the specific hippocampal cell populations involved, the afferent pathways and downstream targets through which microbiome-related signals may influence hippocampal circuits, and the computational variables affected by these interactions.

However, in light of these observations, it plausible that in both health and disease, the gut microbiome participates in cortico-hippocampal predictive coding. This process may be mediated by GABAergic neurotransmission, or more broadly, by E/I balance within cortical circuits, as presented in Figure 1. In hippocampal networks, GABAergic inhibition can contribute to suppressing unwanted and internally generated, intrusive, thoughts. Disruption of GABAergic transmission and the consequent E/I imbalance can therefore impair this inhibitory control, allowing internally generated representations to be misattributed as originating from external sources. This is typically considered a hallmark of psychosis and to result from aberrant inference processes and dysfunctional belief updating. GABA is also considered the computational ‘gatekeeper' of prediction errors. GABAergic networks can modulate prediction error weighting, attenuating error signals when predictions match sensory data and allowing errors to propagate to higher levels of processing when predicted to be precise or informative (Ainley et al., 2016; Edwards et al., 2012; Feldman and Friston, 2010; Peters et al., 2017; Sandved-Smith et al., 2021). Therefore, within this cortico-hippocampal-gastroenteric framework, psychosis could be explained as a consequence—and possibly cause—of dysbiosis, where microbial imbalances in the gut may affect GABAergic regulation of prediction errors in, and by, the hippocampus. This would in turn lead to false inference experienced variously as hallucinations, delusions, agnosia, dissociative syndromes, functional neurological symptoms, and so on.

This article focused on GABAergic transmission for other several reasons. Disruptions in GABAergic transmission are considered very important in the pathogenesis of a range of psychiatric diseases (Marilovtseva et al., 2025). The schizophrenia literature is where the role of GABA in precision-weighting becomes particularly interesting. Schizophrenia has long been associated with aberrant GABAergic neurotransmission (Fogelson et al., 2014; Shaw et al., 2020), and predictive-processing accounts of schizophrenia propose that abnormal excitation–inhibition balance can alter the precision assigned to sensory prediction error signals (Jardri and Denéve, 2013; Friston, 2022). While this article primarily focuses on psychotic manifestations in schizophrenia, similar phenomena can be observed across psychiatric conditions. For instance, decreased GABA transmission was related to the pathogenesis of anxiety and to abnormal processing of aversive prediction errors (Baetu et al., 2018).

Nonetheless, GABA may represent only one of several mediators of the neuro-gastroenteric mechanics of predictive coding and precision-weighting. A comprehensive examination of all potential underlying mechanisms is beyond the scope of the present article. This article aimed to establish a novel conceptual link between gut microbiota and predictive coding through the hippocampus. This, to the best of the author's knowledge, has not previously been articulated, although it builds on existing hypotheses linking microbial imbalances to brain function, to the emergence of psychiatric conditions, and to hippocampal activity. The reader should note that there are other neurotransmitters and neuromodulators produced by the gut microbiota that may play a role in predictive processing. Specifically, dopamine (Hamamah et al., 2022; Millard et al., 2022) and glutamate (White et al., 2015), but also serotonin, acetylcholine and norepinephrine (Chen et al., 2021), although the extent of their contribution to predictive coding remains to be explored. These pathways therefore warrant future discussion and consideration.

Similarly, parallel pathways may operate simultaneously. Dietary choices can affect the gut environment and the production of metabolites that bind to local receptors. The binding would lead to afferent signaling—through, for instance, the vagus nerve—to the hippocampus, affecting GABAergic neurotransmission among inhibitory neurons lower in the predictive coding hierarchy (Barron et al., 2020). The brain can in turn engage descending vagal pathways affecting gut microbiota composition and the production of gut metabolites, in a circular fashion. Moreover, microbiome–gut–brain communication comprises a distributed network of neural, endocrine, immune, and neuromodulatory pathways, engaging brainstem structures such as the nucleus of the solitary tract (NTS), hypothalamic systems, and higher-order cortical and subcortical regions such as the insula, anterior cingulate cortex (ACC), and striatum. The broad framework proposed here focuses on the hippocampus because of its proposed roles in contextual representation, memory-based priors, and precision-weighting, which make it particularly relevant to predictive coding, as considered in this article. However, it is also possible that other brain structures influence predictive coding through their effects on interoceptive or allostatic regulation and prediction (insula, ACC, NTS, Barrett and Simmons, 2015), homeostatic regulation (hypothalamus Migeot et al., 2022), reinforcement learning and prediction error processing (striatum, Schönberg et al., 2007), and prediction error weighting (neuromodulatory systems, Lao-Rodríguez et al., 2026).

Finally, there are conditions under which this framework can be empirically tested or falsified. For instance, if alterations in the microbiota are found not to be associated with corresponding changes in predictive coding; if changes in predictive coding are not accompanied by the predicted involvement of the hippocampus; if hippocampal involvement is epiphenomenal, such that altered hippocampal activity or connectivity does not mediate the microbiome-predictive-coding relationship; if non-hippocampal routes (NTS, hypothalamic systems, insula, ACC, and striatum) provide a more parsimonious explanation of the observed effects. It will also be important to consider that different pathways may be interconnected rather than mutually exclusive, whereby, non-hippocampal brain regions may provide routes through which microbiota-related signals influence hippocampal activity and predictive coding.

## 5 Discussion

### 5.1 Theoretical implications and future directions

This article hypothesized a role for the gut microbiome in predictive coding. Generally, the brain relies on prior expectations about the world (sensory priors) to generate predictions about incoming sensory information. A mismatch between predictions and sampled data creates a prediction error, that is used to update the prior expectations and improve future predictions. In this perspective, the *gut microbiome* is argued to influence how prior *expectations* are processed and *prediction* errors weighed, by the hippocampus. Specifically, predictions are generated from an internal model shaped by prior information, or prior experience, and hippocampal memory representations provide the prior information that informs predictions (Figure 1).

Most importantly, this work adopts the perspective of “mental model” embodiment. Predictive coding is understood with reference to the body, and brain-body causality. Here, this is enacted through interactions with gut microbes. Thus, understanding brain functions requires recognizing that the brain is coupled to different systems in the body. From this perspective, perceptual predictions are embodied and embedded, through interactions with the gastroenteric system and through the ongoing exchanges with the external environment (e.g., provision of food resources). In other words, perceptual processes are not confined to the brain alone, but extend across the body and its environment, including the resources that sustain it. See Bhat et al. (2021) for examples in the immune system and Peters et al. (2017) for discussion in terms of neuroendocrinology and allostatic load.

While it would be interesting to elucidate whether the gut is higher or lower in the hierarchy than the hippocampus, this framework can be considered as a heterarchy, which would also allow for circular causality. Circular causality often attends psychiatric morbidity and, from a computational perspective, the requisite recurrent message-passing implied by predictive coding formulations (Friston et al., 2017; Shipp, 2016). This raises an interesting question: “is the gut part of an embodied generative process, under which predictive processing proceeds?”. To answer this question it can be noted that much of affective inference rests upon “gut feelings” in the form of interoceptive inference (Ainley et al., 2016, 2012; Barrett et al., 2016; Barrett and Simmons, 2015; Seth and Tsakiris, 2018). Furthermore, most formulations of phenomenology turn on the precision weighting mediated by high or deep levels in generative models (Costa et al., 2024; Sandved-Smith, 2025; Sandved-Smith et al., 2021), such as the hippocampus (Barron et al., 2020; Buckner, 2010; Kaplan et al., 2017; Stachenfeld et al., 2017; Whittington et al., 2020).

Important future applications of the proposed cortico-hippocampal-gastroenteric predictive coding framework include guiding (1) the identification of mechanistic biomarkers that characterize patient states—namely, biomarkers related to the regulation of prediction error signals for all, or specific, categories of predictions, regulation of precision weighting of prediction errors, and belief updating—and (2) the selection of personalized dietary interventions, considering the mechanistic relationship between diet and brain health. Specifically, future research could investigate causal relationships and evaluate predictions arising from the proposed hypothesis. Studies should test whether lifestyle interventions (nutrition) can act as model-update drivers and sources of prediction error calibration. These interventions may, indirectly facilitate a shift in brain dynamics away from maladaptive “thought cycles,” or “attractor" states (Adams et al., 2022), and perturb aberrant beliefs, or expectations, according to the clinical presentation of each patient. This will require empirical validation.

Moreover, according to the neuro-gastroenteric mechanism proposed here, hallucinations and delusions in patients with schizophrenia may emerge from reduced microbial metabolite production (GABA), disrupting the hippocampal regulation of GABAergic neurotransmission among inhibitory neurons in the cortical hierarchy. Given that inhibitory synaptic mechanisms determine the precision assigned to sensory prediction errors, an impaired inhibitory control, or neural gain, would result in diminished sensory precision, thereby increasing the relative influence of top-down prior beliefs over incoming sensory evidence. Taken together, this provides a potential mechanistic link to the long-established disinhibition hypothesis (for example see Sterzer et al., 2018), and to the proposed role of GABA in schizophrenia (Glinert et al., 2024). Here, dysbiosis may lead to reduced GABA synthesis and resulting pathophysiology. Nonetheless, it will be important to investigate directional modulations and whether modulating endogenous GABA through nutritional interventions may offer distinct therapeutic effects compared with exogenous GABA administration.

This hypothesis is also in line with accumulating evidence linking dysbiosis to psychosis (Munawar et al., 2021; Tsamakis et al., 2022) and generates experimentally testable predictions, that are as follows. Individuals with psychosis should exhibit altered levels of microbially produced neuroactive metabolites, such as GABA. Microbial alterations should be associated with measures of regional neurotransmitter concentrations—as measured via positron emission tomography (PET), MEGA-PRESS (Peek et al., 2023), proton magnetic resonance spectroscopy, etc.—as well as altered indices of sensory prediction-error processing, such as mismatch negativity (Kirihara et al., 2020; Rowland et al., 2016). Altered microbial metabolite production should predict a shift in perceptual inference toward greater reliance on prior beliefs and reduced weighting of sensory evidence. This hypothesis could be tested by implementing the proposed framework as a dynamic causal model fitted to multimodal patient data. This would allow causal hypotheses about the relationship between dysbiosis, cortical inhibitory signaling, and the precision-weighting of sensory prediction errors to be formally evaluated. Additionally, transferring microbiota with differing metabolite-producing capacity into experimental animal models would help assess effects on cortical inhibitory function and sensory inference. Metabolite supplementation would be expected to attenuate symptoms, providing a direct test of the proposed mechanism.

Most importantly, computational implementations of this mechanistic hypothesis, such as via dynamic causal modeling, should also offer an account of predictive coding abnormalities across other psychiatric conditions (Qela et al., 2025). A comprehensive discussion of these conditions is beyond the scope of this article. Yet, to provide an example, increased variability in attention, impulsive responses, and difficulty maintaining behavioral strategies are typical of ADHD. ADHD has been explained by both gut microbiota alterations (Luyens et al., 2026) and abnormalities in predictive processing (Gonzalez-Gadea et al., 2015). It can also be hypothesized that attentional control and decision-making in this condition may be influenced by deficits in memory integration (Ross and Fernandez, 2026), potentially involving hippocampal–cortical functional interactions. Ultimately, this would result in deficits in perceptual inference, such as increased switching between inward and outward attention, or between maintaining stable predictions and flexibly responding to new sensory data.

From the perspective of psychiatric conditions, precision lifestyle nutrition medicine becomes important, considering patient diversity, differences in individual needs, lived experiences, biological characteristics, behaviors, and environmental contexts. And specifically, considering the substantial inter-individual variability in microbiota composition observed across patients (Kurilshikov et al., 2021). Dietary interventions and nutraceuticals that promote a stable microbiota composition may represent a putative strategy for reducing the risk of developing psychiatric conditions and/or for mitigating symptom severity through the gut–brain mechanisms proposed here. Further work is needed to establish these translational steps in the context of these mechanisms. These steps should include discovery and validation of microbiome-host targets, target-engagement assays and mechanistic randomized trials, to determine the relationship between target modulation and biological and clinical changes. Later stages would also establish efficacy and safety and determine the role for these interventions in clinical practice.

In light of the present hypothesis, it would be especially valuable to use neuroimaging to investigate how GABA-producing bacteria influence brain GABA concentrations and hippocampal neural activity, and to determine whether this pathway represents a potential novel and accessible target. These insights may be valuable for informing the development of targeted nutraceutical interventions for endogenous GABA modulation in psychiatric conditions and for supporting broader aspects of brain health and function. At present, such interventions should be considered as adjunctive approaches to established psychopharmacological treatments and psychotherapy—rather than as primary interventions—pending further research.

### 5.2 Limitations and alternative explanations

There are several confounding factors that may affect the efficacy of dietary interventions. For instance, there are bidirectional interactions between gut microbiota and medications, which are likely to make the development of effective combination therapies more challenging. Psychotropic drugs such as antipsychotics (Cussotto et al., 2019), can influence gut microbial composition and diversity and alter nutrient metabolism, masking or counteracting the metabolic benefits of dietary interventions. In turn, microbial processes in the gut also affect drug metabolism and the absorption of therapeutic compounds. Moreover, exposure to antimicrobial drugs, such as antibiotics and antivirals, can disrupt the composition of gut microbial communities (Zimmermann et al., 2021; Ataei et al., 2026), influencing disease progression and patient responses to dietary interventions. In this context, targeted dietary interventions may, however, contribute to supporting microbiome recovery from medication-induced alterations and the maintenance of a more resilient ecosystem. Ultimately, the above challenges may be mitigated via preclinical testing of medication–microbiome interactions, by considering individual differences in gut microbiota composition, or via strategies that establish a stable baseline microbiota.

Clinical trials of dietary interventions can also be faced by limitations arising from inter-individual and population-level variability. Variability can be observed in metabolic status, body mass index (BMI) and lifestyle factors that influence inflammatory responses and nutrient processing, such as habitual diet, smoking, physical activity, sleep, and stress (Zhou et al., 2025; Healey et al., 2017; Wu et al., 2011; Smith et al., 2019; Sasidharan Pillai et al., 2024; Monda et al., 2017). Moreover, it is important to consider socioeconomic and environmental barriers (Ratcliff et al., 2026), as well as ethnic and geographic variations in gut microbiota composition (Gupta et al., 2017) that are associated with differences in dietary habits. Within patient populations, these sources of variability may be further complicated by gut microbiota changes across disease stages and clinical features (Zhu et al., 2025), as well as by reverse brain-to-gut effects, whereby disease-related changes in the brain influence gut microbiota. Nonetheless, as found for salivary microbes (Qing et al., 2021), gut microbiota alterations that precede disease onset may become important for early disease identification. Mechanistic, or computational, studies are required to establish directionality and causal pathways. These considerations also underscore the importance of conducting personalized screening, of tailoring dietary interventions to individual needs, and of optimizing treatment protocols based on a mechanistic understanding of individual treatment responses.

Finally, there are experimental limitations that may complicate data interpretation, comparison, benchmarking, and translation into clinical applications. For instance, the compositionality of microbiome data, meaning that observed differences in microbiome composition may reflect changes in relative rather than absolute abundance of microbial taxa (Nearing et al., 2021). Additional sources of bias include batch effects arising from differences in sample processing, and functional redundancy in microbial taxa. The latter is important because it contributes to functional resilience, yet it complicates data interpretation, because changes in microbial composition do not necessarily translate into changes in microbial function. Nonetheless, there have been advancements in methodological standardization and efforts to mitigate these limitations (Nearing et al., 2021; Hickman and Korpela, 2025; Bokulich et al., 2020; Ge et al., 2026; Fässler et al., 2025). Finally, there are translational limitations associated with experimental systems, such as human fecal microbiota transplantation to germ-free animal models (Bokoliya et al., 2021). Related findings should be validated in human cohorts, or complemented by computational and mechanistic modeling to further establish causal relationships.

### 5.3 Conclusion

In conclusion, this article provides a unified hypothesis, whereby the gut microbiota influences the hippocampal-dependent modulation of hierarchical predictive coding, such as the encoding of priors as memory representations and the precision weighting of prediction errors that drive learning and the updating of prior expectations. This article also underscores the importance of mechanics-informed and personalized dietary and nutraceutical interventions, moving beyond the “one target, one treatment” philosophy.

## Data Availability

The original contributions presented in the study are included in the article, further inquiries can be directed to the corresponding author.
